# Multistage Digital-to-Analogue Chip Based on a Weighted Flow Resistance Network for Soft Actuators

**DOI:** 10.3390/mi12091016

**Published:** 2021-08-26

**Authors:** Zhou Zhou, Manman Xu, Chenlin Zhu, Gonghan He, Kunpeng Zhang, Daoheng Sun

**Affiliations:** 1 Department of Mechanical Engineering, Anhui Ploytechnic University, Wuhu 241000, China; xumanman@ahpu.edu.cn; 2 Department of Mechanical Engineering, China Jiliang University, Hangzhou 310000, China; zhuclgary@cjlu.edu.cn; 3 Shenzhen Research Institute of Xiamen University, 19th Gaoxin South Fourth Road, Shenzhen 518000, China; hgh@xmu.edu.cn; 4 Fujian Engineering and Research Center of Intelligent Sensing and Control for High-End Equipment, Xiamen University, Xiamen 361000, China; xmuzhkp@xmu.stu.edu.cn (K.Z.); sundh@xmu.edu.cn (D.S.)

**Keywords:** microfluidic, soft robotics, hydraulic actuators, analog circuits

## Abstract

A control chip with a multistage flow-rate regulation function based on the correlation between the flow resistance and flow rate has been developed in this article. Compared with the traditional proportional solenoid valve, this kind of flow valve based on microfluidic technology has the characteristics of being light-weight and having no electric drive. It solves such technical problems as how the current digital microfluidic chip can only adjust the flow switch, and the adjustment of the flow rate is difficult. To linearize the output signal, we propose a design method of weighted resistance. The output flow is controlled by a 4-bit binary pressure signal. According to the binary value of the 4-bit pressure signal at the input, the output can achieve 16-stage flow adjustment. Furthermore, we integrate the three-dimensional flow resistance network, multilayer structure microvalve, and parallel fluid network into a single chip by using 3D printing to obtain a modular flow control unit. This structure enables the microflow control signal to be converted from a digital signal to an analogue signal (DA conversion), and is suitable for microflow driving components, such as in microfluidic chip sampling systems and proportional mixing systems. In the future, we expect this device to even be used in the automatic control system of a miniature pneumatic soft actuator.

## 1. Introduction

The fluid signal processing function has been one of the most popular research directions for microfluidic devices in recent years. It is also a significant branch of digital microfluidic technology. Compared to being used as an experimental container for biochemical reactions, a microfluidic device with this function can expand the application of microfluidic technology to mechanical and electronic fields. As early as 2015, Au et al. reported the prototype of a microfluidic device with automated control functions [1]. Subsequently, based on summarizing the microfluidic logic control methods in recent years, Sochol et al. reported a microfluidic device with a digital processing function fabricated through a 3D printing method [2] that can process fluid signals itself. Woodhouse et al. demonstrated a microfluidic logic system and applied it to the study of the self-assembly behavior of photochemically active matter [3]. Elatab et al. noted that the next generation of microfluidic devices should be able to perform in situ analysis and processing of complex fluid behavior, similar to the current integrated electronic circuits that use logic gates to perform complex operations. This would enable the microfluidic system to make decisions autonomously according to Boolean rules and eliminate the need for any external intervention [4]. Some emerging fields have urgent needs for this technology. For example, Wehner et al. reported that soft robots have many attributes, many of which will be very complicated when implemented with rigid structures. The soft robot must be bound to the control and power systems of the rigid robot. To realize the potential of soft actuators, new strategies are needed to create completely soft drives, actuators, and robot structures. These key components include flexible mechanical systems and pressure-driven 3D microfluidic chips with logic operations [5]. Pneumatic soft actuators can contact and manipulate soft and fragile objects without damaging them, such as manufacturing assembly line robots, automatic packaging robots, fruit picking robots, and so forth. Furthermore, it can safely interact with human beings, which is suitable for nursing workplaces, such as hospitals and nursing homes. Its continuous working mode is also similar to the working process of bionic motion robot muscles, which has great bionic application potential.

At present, fluid signals, such as flow and pressure, can be processed by microfluidic devices, but the methods are mainly based on switching behaviour. The basic element is the membrane microvalve structures invented by Quake [6]. On this foundation, researchers have realized a series of logic behaviours that achieve functions similar to electrical systems. This kind of chip is also called an integrated fluidic circuit (IFC) [7]. These devices are based on fluid equivalent theory [8]. Under laminar flow, the voltage-resistance-current relationship in the electronic circuit approximately corresponds to the pressure-flow and resistance-flow-rate relationship in the fluidic circuit. By transforming the diodes in the electronic circuit into microvalves in the fluidic circuit, the microfluidic chip itself can perform direct logic control based on the fluid motion behaviour and realize the conversion from ‘electronic-controlled fluid’ to ‘fluid-controlled fluid’. The logic function of electronic circuits can be correspondingly used in the microfluidic chip [9]. Lesher–Perez et al. used the phase difference between multiple microvalves to realize fluidic oscillation behaviour, and the output could be oscillating pressure or flow rate signals [10]. The structure was similar to the ring oscillator in an electronic circuit, and the generated pressure or flow rate signal were used in the drive of a vibrating table [11] and a soft actuator [5]. Grove et al. used vacuum pressure as the source signal to obtain the logic gate structure of AND/OR/NO. These gates can be connected into a high-order digital network to realize the trigger and latch structure under a fluid medium. These devices realize the signal storage function of the chip by the microfluidic system itself [12]. Zhou et al. proposed the idea of a microfluidic arithmetic unit and carried out the preliminary design and experiment of the adder logic [13]. Although IFC chips have achieved certain research results, their functions still belong to the category of digital circuits in electronics. The signals of their output ports are all with or without fluid, and the difference in signals is only distinguished by the difference in the flow channels. According to the history of the electronics field, this pure digital signal processing has limited development potential. Digital circuits always need to be used in conjunction with analogue circuits to enrich the control functions [14].

In a microfluidic system, we can regard analogue signal processing as the continuous physical expression of the flow rate or pressure, in other words, as control of the flow rate or pressure. Most microfluidic device functions are realized based on flow rate regulation, such as the reaction rate of polymerase chain reaction [15], physical sorting performance of cancer cells [16,17], noise and thermal response performance of an enzyme measurement sensor [18], and dynamic dialysis speed of a solution [19]. Normally, we can easily adjust the flow rate in a pipeline through manual control, such as via faucets and drain valves. However, in the field of automatic control, there is no strict analogue signal. The analogue signals we currently use are processed from digital signals. To obtain an analogue signal, a device with a digital-to-analogue (DA) conversion circuit must be used. Therefore, the current flow adjustment in a microfluidic system still relies on external electromagnetic devices, such as syringe pumps or peristaltic pumps, or on variable physical fields such as electricity, magnetism, and heat. These external devices and physical field controllers must also have analogue output. Heo et al. began with the pump control algorithm and improved the accuracy and stability of the flow regulation in a microfluidic network [20]. Xu et al. used the adjustable temperature to change the size of vapour bubbles in a flow channel and blocked fluid movement through the bubbles, which can realize flow adjustment [21]. Zhang et al. used a membrane microvalve as a flow adjustment unit and adjusted the output flow by changing the opening size of the valve core through adjustable air pressure [22]. Perdigones et al. reduced the stiffness of a membrane microvalve and achieved a larger range of flow regulation under the same external pressure [23]. Casals–Terré et al. set up a V-type cobalt-nickel electrodeposited layer in a flow channel. An external permanent magnet field drives the movable structure attached to the deposition layer and changes the size of the flow channel opening to achieve the flow rate regulation function [24]. Johansson et al. used MEMS technology to make a silicon-based piston microvalve structure to adjust the gas flow [25]. Chong et al. used the acoustic induction method to change the flow rate in a T-shaped channel, which can adjust the size of the generated droplets [26]. To date, no microfluidic chip can adjust the flow rate without external analogue control signals.

In this paper, we report a microfluidic system based on a weighted flow resistance network that converts multichannel pressure digital signals to an adjustable flow rate. Its design and manufacturing process are simple, and it can be easily integrated into existing microfluidic devices. In the current design, the output flow rate can be adjusted via the permutation and combination of the pressure signals at the four inlets, which are either with or without pressure. This is similar to the conversion of binary numbers to decimal numbers. We demonstrate the concept of weighted flow resistance. By weighting the flow resistance of different branches, we can obtain the upper limit of the flow rate that can be controlled by each branch. We use membrane microvalves to control the ‘on’ and ‘off’ state of each branch. The control signals of these membrane microvalves are the 4-bit pressure signals we input. When the input signal is 4 bits, the output can realize 16-level orderly control. According to experiments, this microfluidic chip with a DA conversion function shows good linearity in its flow curve. Table 1 summarizes the characteristics of different flow regulation methods. In this paper, the flow control method with a DA conversion function provides novel ideas for the application of microfluidics in the field of pneumatic or hydraulic automatic control.

## 2. Materials and Methods

Signal weighting and pulse modulation are two main methods of DA conversion in electronic systems. A weighted resistance network, which has similar characteristics to a fluid network, is used to design the logic network. We define it as the weighted flow resistance network.

**Figure 1:**
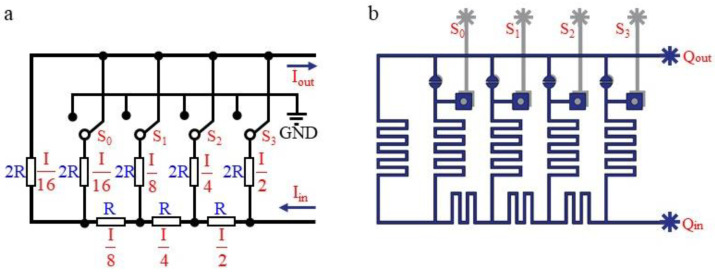
Principle of the flow regulation chip: (**a**) Electronic circuit model of DA conversion; (**b**) equivalent model of the weight flow resistance for flow rate regulation.

An electrical schematic diagram of the weighted current resistance network is shown in Figure 1a. For the convenience of illustration, only four parallel branches are selected. As a parallel system, regardless of the states that switches S_0_–S_3_ are in, the current through each branch is obviously constant. Because the parallel branches have different resistances, the current will be weighted according to the different branches. For example, when the total flow rate of the trunk channel is I, the current of branch S_3_ is I/2, S_2_ is I/4, S_1_ is I/8, and S_0_ is I/16. These weighted currents will be collected at the outlet end for the addition operation to obtain the total outlet current. Therefore, if the current in a branch is grounded, then the current in that branch cannot be added at the outlet, and the current at the outlet will be reduced. The two position states of switch S_n_ can be regarded as binary 0 or 1. Due to the different weights of each branch where Sn is located, four switches can form a 4-bit binary number to represent the output flow. This is the basic principle of weighted resistance. The fluid network under this principle is shown in Figure 1b.

**Figure 2:**
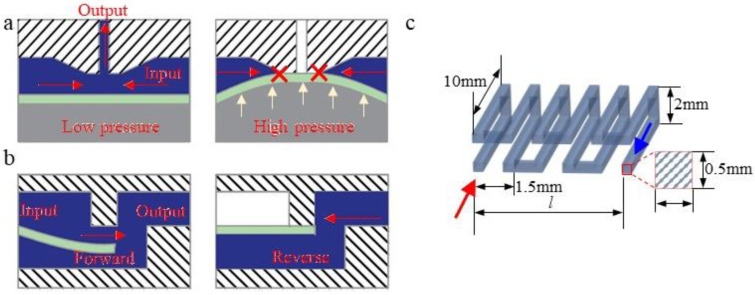
Principle of microvalves and flow resistance: (**a**) Structure of the normal open membrane microvalve; (**b**) Schematic diagram of check valve; (**c**) Structure of the 3D flow resistance.

In a fluid network, the switch operation function can be realized by membrane microvalves. As shown in Figure 2a,b, the microvalve used in this paper is a circular three-layer structure with a diameter of 5 mm; the bottom layer is the control layer, the middle layer is a flexible PDMS membrane, and the upper layer is the working layer. When pressure is applied to the control layer, the membrane deforms upwards and closes the flow channel in the device layer to achieve the closing function [27]. The microvalve is not connected in series in the branch, but in parallel with the flow resistance in the branch, and connected to the atmosphere. The microvalves are always in the open state under normal conditions. Then, most of the flow in the branch flows out through the microvalve. At the final output port, the weight of the branch flow contribution is almost zero. The microvalve is closed when a loading pressure is applied at the control layer. At this time, the fluid in the branch will converge on the output port, increasing the flow rate of the output port.

To characterize the analogue quantity, we define the following: 0 means that the membrane of the microvalve is not subjected to pressure and the microvalve is in the open state; and 1 means that the membrane is subjected to pressure and the microvalve is in the closed state. The numerical value and sequence of the four microvalve working states are expressed as a four-digit binary number, S_3_S_2_S_1_S_0_. Obviously, this number sequence has 16 different input states ranging from 0000 to 1111. According to the law of DA conversion, if the input flow is Q, then the value of the binary number increases by 1, and the corresponding output flow increases by Q/16. Since there are microvalves in each branch to communicate with the atmosphere, we need to ensure that the final output trunk fluid does not flow back to other branches with a state of 0. Therefore, we set up a membrane check valve at the end of each branch. This kind of check valve has a hole on one side of the membrane, so the fluid has different pressure characteristics for the membrane when the fluid flows in the forward and reverse directions, similar to the function of a diode in an electronic circuit [28].

The flow resistance is a property that directly causes different flow rates in each branch. The flow resistance in microfluidics is usually related to the cross-sectional size and length of the flow channel and to the existence of local vortices. Generally, if a flow channel with a small cross-section is adopted, then there must be a large gap between the flow resistance scale and the scale of the other flow channels. This multiscale processing is unrealistic with current processes. The flow resistance in a direct flow channel is small, but such a channel requires a large amount of chip space. The local vortices are obviously affected by the flow rate, especially in microscale channels, which will cause the flow resistance to exhibit different flow resistance characteristics under high and low flow input. To solve these problems, the flow resistance in this paper is designed based on a three-dimensional tortuous structure, in which each inflection point of the flow resistance can produce a certain vortex, and this three-dimensional structure is also conducive to the growth of the flow channel size. The structure and size of the flow resistance network are shown in Figure 2c.

**Figure 3:**
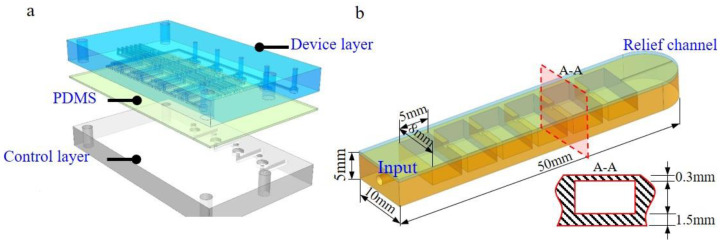
Model of the chip and soft actuator: (**a**) Integrated chip with 3 layers; (**b**) soft actuator with comb tooth cavity.

In the entire weighted flow resistance network, we adjust the resistance by changing the axial length l of the flow resistance network, where the length is related to 2R. We arrange these three-dimensional structures according to the network diagram of the desired flow resistance and obtain the structure model of the microfluidic chip shown in Figure 3a (Supplementary Material for detailed structure). For the convenience of manufacturing and maintenance, the chip has a three-layer assembly structure, which is equipped with screws and nuts. The three layers are fixed tightly. Among them, the upper and lower layers are the device and control layer structures, and the middle layer is a PDMS film. A hose can be directly inserted into the interface, sealed and fixed with UV glue.

To visualize the flow regulation, we connect this flow regulation chip with a flexible actuator structure. The actuator is shown in Figure 3b. This flexible actuator can be understood as having a high flow resistance in the series flow channel. It contains an elastic cavity with a low flow resistance and a pressure relief channel with an extremely high flow resistance. The pressure relief channel is located at the end of the cavity and is obtained by puncturing the silicone structure with a 150μm needle after the moulding is completed. When a certain flow flows through the flow resistance network, a pressure difference will appear at both ends of the flow resistance network, which drives the elastic structure to bend. When the digital flow control chip inputs different flow rates, the actuator will bend at different angles. The input port structure of the actuator structure is made by cutting out the silica gel with a punch.

## 3. Process

Soft lithography and laser etching are classical methods for processing microfluidic chips, but are only applicable to two-dimensional planar structures. The device in this paper contains a flow resistance network with three-dimensional features. When soft etching or laser technology is used, multilayer slicing processing and layer-by-layer bonding must be performed. Not only is this process extremely difficult, but the sealing performance of the device is also easily reduced. Therefore, we chose 3D printing to realize the device. A Projet 3600 printer (3D Systems, Rock Hill, SC, USA) that uses the principle of Multi-Jet Printing (MJP) for structural moulding is used. Its support material is paraffin, which can be removed by soaking in a solvent. This has been proven to be a feasible 3D printing method for manufacturing microfluidic chips [2]. The VisiJet M3 Crystal material (3D Systems, Inc. Rock Hill, SC, USA) is a transparent plastic, and its mechanical properties are similar to those of PMMA plastic. The PDMS membrane is made by a spinning coating process. Because the scale of the 3D printing device (millimetres) is larger than that of soft etching (micrometres or nanometres), the device in this article does not require a particularly thin PDMS membrane. During processing, the speed of the homogenizer is 300 rpm, and the time is 30 s. We obtain a 100 μm thick PDMS film. The holes on the film are first positioned by laser marking, with ablated small black spots indicating the position, and then punched with a circular punch. Due to the roughness of the 3D printed surface, bubbles and air leakage are prone to occur at the interface of the 3D printing material and PDMS. We sprayed a layer of liquid adhesive (3M, Inc. Saint Paul, MN, USA) on the surface of the PDMS film before assembling the structure to ensure tightness. To avoid thermal deformation, the entire manufacturing process is completed at room temperature to complete the assembly and glue curing process. Furthermore, we used M3 threads to pre-tighten the connectors.

The chip and the hydraulic source are connected with a flexible tube that is fixed with UV glue. As the size tolerance of the 3D printed circle is difficult to control, we used a standard drill to perform post-processing, achieving an interference fit. By adjusting the amount of interference, the maximum pressure that this connection method can withstand in the experiment is approximately 500 kPa, which fulfils the test requirements (for safety reasons, we limit the input pressure to less than 100 kPa).

Silicone material (Aladdin, Inc. Shanghai, China) is used to mould the soft actuator. The mould is made by 3D printing (Dimension Elite, Stratasys). To ensure the feasibility of the moulding process and the tightness of the cavity, the structure is designed with two layers: one is the cavity, and the other is the surface sealing film. The cavity has a comb-like structure to realize anisotropic mechanical properties of the elastic cavity. The elastic film is obtained by spin coating and has a thickness of approximately 300 μm. The two layers are tightly connected by semi-cured bonding. First, a silicone film (glass substrate, 500 rpm/min) is spin-coated. The film is cured at room temperature for 15 min to obtain a semi-cured silica gel film. Then, the structure layer with the cavity is gently placed on the surface of the film and cured at room temperature for 2 h to obtain a flexible actuator structure with feasible airtightness.

The microvalves can be driven by air pressure or hydraulic pressure. To facilitate the experiment, we chose pneumatic drive, and the air pressure source was a gas cylinder. The output pressure of the gas cylinder was adjusted, and the gas was input into the chip input port through four parallel branches. The input ports of S_0_–S_3_ were individually connected to solenoid switch valves to generate 0/1 signals of the input ports. These solenoid valves are controlled by an Arduino Uno microcontroller. The flow input port of the chip was connected to a syringe pump to ensure a constant flow at this port. The output end of the chip is directly connected to the atmospheric environment after passing through a flow sensor, and real-time flow signals are obtained through computer collection. In this experiment, the lower layer of the micro valve adopts air pressure input, and the fluid medium connected with Q_in_ port is deionized water. The experiment was carried out at room temperature of 25 °C and humidity of 60%. The precision flow sensor flow unit (Fluigent, Inc. France) is used for flow monitoring and data acquisition.

## 4. Results and Discussion

**Figure 4:**
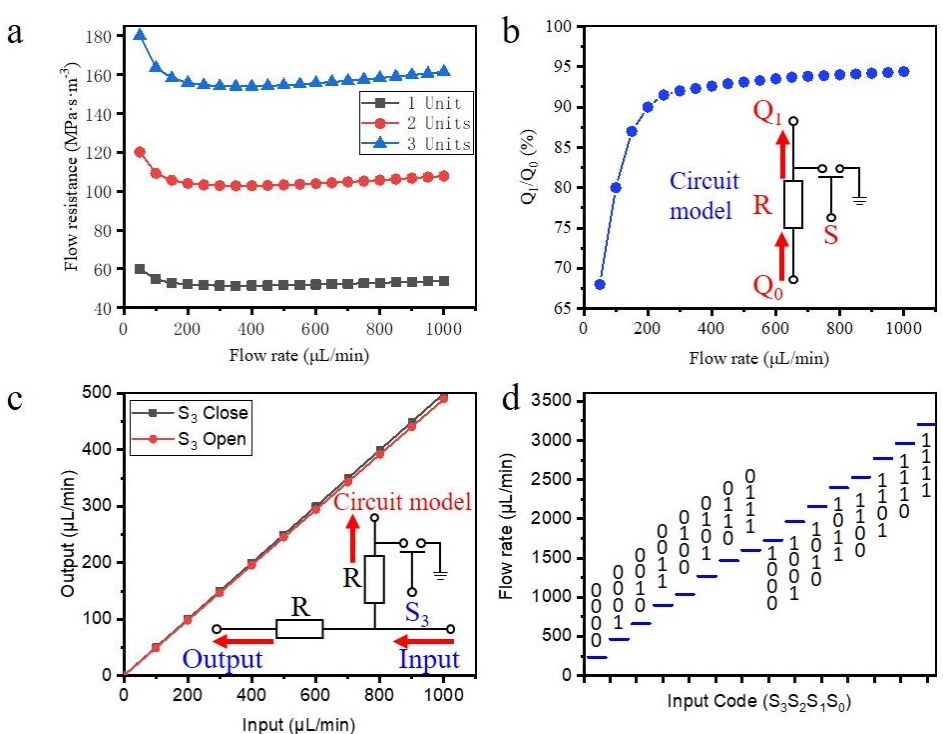
Simulation results of flow control chip. (**a**) The flow resistance curve under different flow rates and different flow channel lengths; (**b**) Flow rate in the branch output port when the microvalve of this branch is opened; (**c**) Mutual interference curve of the flow rate between different branches; (**d**) The simulation curve of input and output characteristics during the working process of the entire system.

The software used for simulation analysis is Comsol Multiphysics (Comsol, Inc. Stockholm, Sweden). The grid is a hexahedral grid with five boundary layers, and the minimum mesh size is 20 μm. The inlet is a constant flow rate boundary, and the outlet is an open boundary. The flow resistance can be expressed as R = Q/Δp, where Q is the inlet flow rate and Δp is the pressure difference between the two ends of the flow resistance network. Figure 4a shows the change in flow resistance under different flow rates and different flow channel lengths, where the length of a single unit is l = 3 mm. The simulation results show that the flow resistance in the flow channel changes nonlinearly with increasing flow rate. When the flow rate changes from 0 to 1000 μL/min, the flow resistance has a change rate of approximately 20%. With a further increase in the flow rate, the change curve of the flow resistance gradually becomes a smooth, straight line. When the input flow rate remains steady, the flow resistance has an obvious linear relationship with the length of the flow channel. These results indicate that the input flow rate does not have a very large impact on the flow resistance, and the flow resistances for different lengths are proportional under the same flow rate. For the design dimensions in this article, when the flow rate is less than 200 μL/min, the rate of change in the flow resistance is relatively large. When the flow rate is higher than 200 μL/min, the flow resistance change curve is basically level. Therefore, to ensure the consistency of the flow resistance in each branch of the device, we need to ensure that the smallest flow rate is greater than 200 μL/min, that is, Q/16 = 200 μL/min, where Q is represented by the minimum flow rate for the binary number 1111.

To reduce the amount of calculation, subsequent simulations use two-dimensional models for calculation, and the flow resistance values in these two-dimensional models are consistent with those in the three-dimensional models. Figure 4b shows the flow rate in a branch when the branch microvalve is opened. This analysis is mainly employed to ensure that the branch microvalve can function as a diversion channel. The simulation results show that when the microvalve is opened, 95% of the flow in the branch can be discharged through the microvalve. Due to the adhesive force and surface tension of the fluid itself, part of the fluid in the branch will always enter the output channel. However, because the amount is very small, it is temporarily ignored here.

Figure 4c shows the mutual interference curve of the flow rate between different branches. During the simulation process, microvalve S_3_ is controlled to switch between 0 and 1, and the output flow rate for the flow resistance R in parallel with the S_3_ branch is tested. The flow through R directly enters the follow-up branch. The calculation results show that the input flow of the subsequent branch changes with the change in the S_3_ state, but this change is only approximately 2%, so the influence of a single branch microvalve on the remaining branches can be ignored.

Figure 4d shows the simulation curve of the input and output characteristics during the working process of the entire system, where the binary sequence of the input port is {S0, S1, S2, S3} = 0000~1111. The simulation results show that the system can achieve 16-level flow regulation. The flow rate of each sequence in order shows a linearly increasing regulation.

**Figure 5:**
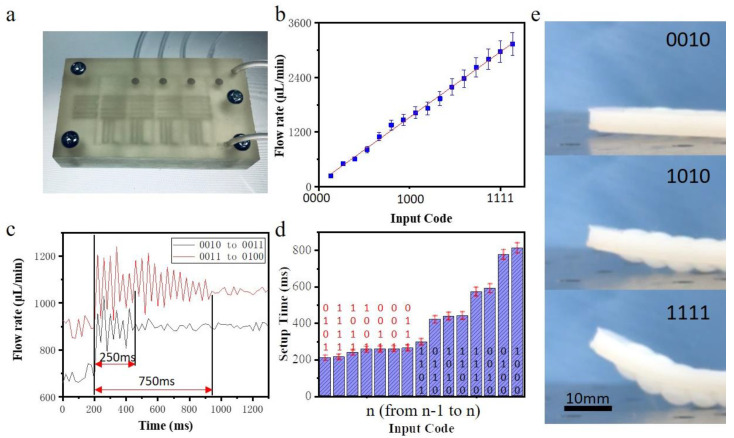
The DA chip operation. (**a**) Three-dimensional printed digital-analog chip; (**b**) The flow regulation curve of the device, input flow rate is 5000 μL/min; (**c**) The curve of setup time of code 0010 to 0011 and 0011 to 0100; (**d**) Setup time of each convention stages; (**e**) A soft actuator driven by the DA chip.

Figure 5a shows the experimental device obtained by 3D printing. Although the material itself is of natural colour and low transparency, the three-dimensional channel and microvalve structure are still clearly visible. Figure 5b shows the flow regulation curve of the device when the input flow is 5000 μL/min. Experiments show that the device basically achieves 16-level flow regulation, but its linearity is lower than the simulation results. Next, we test multiple batches of devices. The linearity error is approximately 5%, indicating that the linearity difference is mainly caused by the inherent error of the manufacturing method. The 3D printing method leads to variations in roughness and microstructure. Much room for improvement remains in terms of consistency. The settling time is an important parameter used to describe the speed of DA conversion, and it can be equivalent to the stabilization time when the control signal is switched. We increase the sampling frequency of the flow signal to 50 Hz and roughly measure this physical quantity through simple waveform analysis. As shown in Figure 5c, during the different stages of DA conversion, obvious differences are observed in the settling time. For example, the settling time is approximately 250 ms from 0010 to 0011, but the settling time is as long as 750 ms from 0011 to 0100. Figure 5d shows the settling time for each conversion stage from n-1 to n, where n is the input binary number. The result shows that the settling time of DA conversion is related to the number of microvalve switch state conversions in each stage. For example, only one microvalve is involved in the 0010–0011 process, while two microvalves have their states flipped in the 0011–0100 process. When more microvalves participate in DA conversion, the settling time will be longer.

When a certain flow passes through a high flow resistance network, a large pressure difference will be generated at both ends of the flow resistance network. This pressure can be used to drive flexible actuator movement. Figure 5e shows the movement of a flexible bending actuator driven by the flow regulation. The flow rate at the input end of the device is 5000 μL/min for the working conditions of the flexible manipulator when collecting different binary input signals. As the input binary signal increases, the bending of the flexible manipulator gradually increases.

## 5. Conclusions

This article investigated the structure and performance of a microfluidic DA conversion device that uses the design approach of an electrical circuit. The conversion unit can operate with four input pressure codes and achieve 16-stage output flow rates. The experiments demonstrate that this device can realize continuous multistage flow rate adjustment. The basic device-operating characteristics have been obtained by signal analysis, with a 250~750 ms settling time and a 5% linearity. By connecting the microfluidic chip to a pneumatic flexible actuator, we simply demonstrate an application of the DA chip in the driver of a soft robot. The associated modelling allows us to control a complex microfluidic system with only a pump, and not only channel switch operation, but also flow rate adjustment. In the future, such microfluidic processors will have the potential to replace electronic devices in certain special environments, such as underwater environments or those with strong electric or magnetic fields.

## Figures and Tables

**Table 1:** Summary of the characteristics of different flow regulation methods.

Serials	Parameter	Flow Control Unit	Feature Size	Steps of Flow Rate	Technical Features
Ref [20]	Pressure amplitude	Flow resistance	100 μm (width)	6	PID control
Ref [22]		Membrane valves	150 μm (width)	With the steps of input pressure	Film deformation
Ref [23]		Membrane valves	1 mm (diameter)		
Ref [25]		Wing-shaped flow restrictor	200 μm (width)	continuous (noliner)	MEMS technology
Ref [21]	Heating temperature	Vapor valves	150 μm (width)	Continuous	Two-phase flow interaction is used for regulation
Ref [24]	Magnetic field	Magnetron membrane valves	3 mm (diameter)	6	Controlled by magnetic field
Ref [26]	Acoustic frequency	Y type channel	100 μm (width)	Continuous	Characterized by two-phase flow droplet size
This paper	On/off of the pressure	Membrane valves and flow resistances	500 μm (width)	16	Flow resistance network

## Supplementary Materials

The following are available online at https://www.mdpi.com/article/10.3390/mi12091016/s1.
